# Microorganisms and Mortality Factors in Hospitalized Hemodialysis Patients with Catheter-Related Bloodstream Infection and Infective Endocarditis: 7 Years of Experience

**DOI:** 10.3390/jcm15051815

**Published:** 2026-02-27

**Authors:** Feyza Bora, Umit Cakmak, Özlem Esra Yıldırım, Funda Sarı

**Affiliations:** 1 Department of Internal Medicine, Division of Nephrology, Akdeniz University Faculty of Medicine, Antalya 07059, Türkiye; 2 Department of Internal Medicine, Division of Nephrology, Antalya Memorial Hospital, Antalya 07025, Türkiye; 3 Department of Internal Medicine, Torul State Hospital, Gümüşhane 29800, Türkiye

**Keywords:** hemodialysis, catheter-related bloodstream infection, infective endocarditis, *Staphylococcus aureus*, mortality, echocardiography

## Abstract

**Background and Objectives:** Catheter-related bloodstream infections (CRBSIs) and infective endocarditis (IE) lead to substantial morbidity, prolonged hospitalizations, and increased mortality. This study aimed to determine the incidence of IE among hospitalized catheter-dependent HD patients with CRBSI and identify risk factors associated with 90-day all-cause mortality. **Materials and Methods:** We conducted a retrospective analysis of patients diagnosed with CRBSI. Clinical, microbiological, and accessible echocardiographic data were evaluated. Risk factors for 90-day mortality were analyzed using univariate analysis and multivariable binary logistic regression analysis models. **Results:** A total of 85 hospitalized catheter-dependent HD patients with CRBSI were included. Gram-positive organisms were the predominant pathogens (70.6%), with *Staphylococcus aureus* identified in 35.3% (30/85) of all CRBSI cases. Gram-negative bacteria accounted for 29.4% of all CRBSIs. IE was identified in 9.4% (*n* = 8) of patients diagnosed with CRBSI. Significant differences were observed between the IE and non-IE groups regarding the need for length of hospital stay, vegetation, embolism (*p* < 0.05). The 90-day all-cause mortality rate was 14.1% (*n* = 12). Univariate analysis identified that older age and female gender were associated with increased mortality (*p* < 0.05). In the multivariable binary logistic regression, only age (OR: 1.055, 95% CI: 1.005–1.107, *p* = 0.029) remained an independent predictor of 90-day mortality. **Conclusions:** In catheter-dependent HD patients, *Staphylococcus aureus* is the predominant organism associated with both CRBSI and IE. With an observed IE occurring in 9.4% hospitalized catheter-dependent HD patients with CRBSI, consistent compliance with prevention bundles must be prioritized as a standard of care for catheter management.

## 1. Introduction

Catheter-related bloodstream infections (CRBSIs) remain a formidable challenge in maintenance hemodialysis (HD), significantly escalating morbidity and healthcare costs [1]. Patients undergoing maintenance HD often necessitate prolonged central venous catheter (CVC) dependence, which is inherently associated with an elevated risk of CRBSI [2]. CRBSI typically arises via extra or intraluminal colonization, which is often dictated by the duration of catheter placement. Catheters inserted for short periods (e.g., less than 7 days) are frequently colonized via the intraluminal route, typically originating from hub contamination [3].

CRBSI in the chronic HD population is a critical public health concern, consistently linked to substantial increases in morbidity, prolonged hospitalization, and premature death [4]. According to a meta-analysis conducted in 2024, the incidence of CRBSI in hemodialysis patients is driven by a complex interplay of clinical and procedural determinants. Key risk factors include the use of high-risk non-tunneled temporary catheters, suboptimal insertion sites—with the femoral vein posing the highest risk, followed by the internal jugular, while the subclavian vein remains the least risky—and prolonged catheter duration alongside frequent catheterizations. Patient-specific vulnerabilities such as advanced age, a history of prior catheter-related infections, and long-term dialysis vintage further exacerbate the risk. Furthermore, physiological and biochemical markers, including low albumin, lower hemoglobin, and reduced CD4+ T-cell counts, combined with comorbidities like diabetes mellitus, hypertension, and high APACHE II scores, are significantly associated with increased infection rates. Finally, poor adherence to hand hygiene protocols remains a critical, modifiable factor in preventing these bloodstream infections [5].

Recent epidemiological evidence demonstrates a substantial difference in CRBSI rates based on catheter type in hemodialysis patients. Research indicates that temporary non-tunneled catheters are associated with substantially higher infection rates compared to tunneled catheters, ranging from 0.48 to as high as 16.85 per 1000 catheter days, particularly when placed in the femoral vein, where risks can escalate to 20.2 per 1000 days [6,7,8]. In contrast, tunneled cuffed catheters generally exhibit lower incidence rates, typically between 0.48, 2.9 and 15.14 per 1000 catheter days, due to the protective dacryon cuff that serves as a barrier to bacterial migration [6,7,8]. A landmark meta-analysis confirms that catheter-reliant patients face a 1.5-fold increase in all-cause mortality and a more than two-fold increase in fatal infection risk compared to those with fistulas [9]. The predominant causative agents for CRBSIs are Gram-positive bacteria, which constitute up to 80% of cases and primarily include *Staphylococcus aureus* and *Coagulase-negative staphylococci* [10,11,12].

The primary cause of IE is the pathogenic infiltration of the endocardium by microorganisms, including bacterial and fungal species. The clinical consequence of this invasion is characterized by rapid destruction of the heart valve or the delicate lining of the ventricular wall. The incidence of IE in HD patients is reported to be between 2% and 5%, with recent studies indicating an alarmingly high mortality rate of 41.3% to 56 [5,13]. *Staphylococcus aureus* is also the predominant causative organism in this setting, responsible for 37% to 65% of IE cases [14].

Despite the established high risk of IE in HD patients, regional variations in microbial spectrum and clinical outcomes necessitate continuous surveillance. The objective of this study was to evaluate the incidence of IE among catheter-dependent HD patients with CRBSI, and to identify the risk factors associated with 90-day all-cause mortality in this hospitalized population.

## 2. Materials and Methods

### 2.1. Patient Selection Criteria, Study Design

This retrospective study utilized data from hospitalized catheter-dependent HD patients at the nephrology department of Akdeniz University Hospital, a tertiary academic medical center serving a population of 2.7 million inhabitants in Antalya, Turkey. Catheter-dependent HD patients with suspected CRBSI were enrolled from both various surrounding centers and our own in-center unit. Comprehensive clinical data were extracted from electronic medical records using a standardized data collection form.

### 2.2. Study Participants

Patients were identified by searching the database for all admissions from January 2016 to December 2021. Patients referred from external centers were admitted either through the emergency department or the nephrology outpatient clinic. Patients admitted to the nephrology inpatient service between the specified dates were individually examined, and those evaluated by the infectious disease specialists and diagnosed with Central Line-Associated Bloodstream Infection (CLABSI) using the CDC/NHSN definition were included in the study for screening. This study defines a suspected case of CLABSI as a patient with an indwelling dialysis catheter who presents with signs and symptoms of fever (>38 °C) or chills with no other obvious source of infection [15]. Data from patients hospitalized with laboratory-confirmed CLABSI were analyzed to identify candidates. CRBSI diagnosis was established according to Infectious Diseases Society of America (IDSA) guidelines; only those meeting the criteria for definite or possible CRBSI were eligible for the final analytic inclusion [16]. Definite or possible IE diagnosis has been based on the 2023 Duke-International Society for Cardiovascular Infectious Diseases Criteria for Infective Endocarditis (ISCVID) criteria [17]. The catheters were used solely for hemodialysis purposes. It is unknown whether or not they have non-functioning AVFs. They have no AVF grafts. Definite and possible CRBSI ((IDSA) guidelines) and definite and possible IE (2023 Duke–ISCVID) criteria are explained in Appendix A.

### 2.3. Data Collection

Demographic information, duration and type of indwelling catheter, comorbidities (diabetes mellitus, hypertension, immunosuppressant use, congestive heart failure), predisposing cardiac conditions, causative organisms, laboratory results, echocardiographic findings, complications, length of hospitalization, 28- and 90-day all-cause mortality were recorded. Transthoracic echocardiography (TTE) was performed in 69 of the 85 patients (81.2%). Due to the retrospective nature of this study, the absence of echocardiographic data in certain cases may be attributed to a low clinical suspicion of infective endocarditis, particularly in patients who exhibited rapid clinical recovery and a significant decline in inflammatory markers. Furthermore, logistical challenges in scheduling examinations during the hospitalization period may have also contributed to the incomplete data. Transesophageal echocardiography was utilized when deemed necessary. Patients who underwent TTE were evaluated for the presence of vegetation, cardiac function indices, and pulmonary artery pressure values. In addition, extracardiac complications such as emboli to vital organs were documented. While 28-day mortality primarily reflects the acute severity of the infection and the immediate efficacy of the initial antimicrobial therapy or surgical intervention, 90-day mortality serves as a more comprehensive indicator of the long-term survival and the patient’s overall resilience.

**Figure 1:**
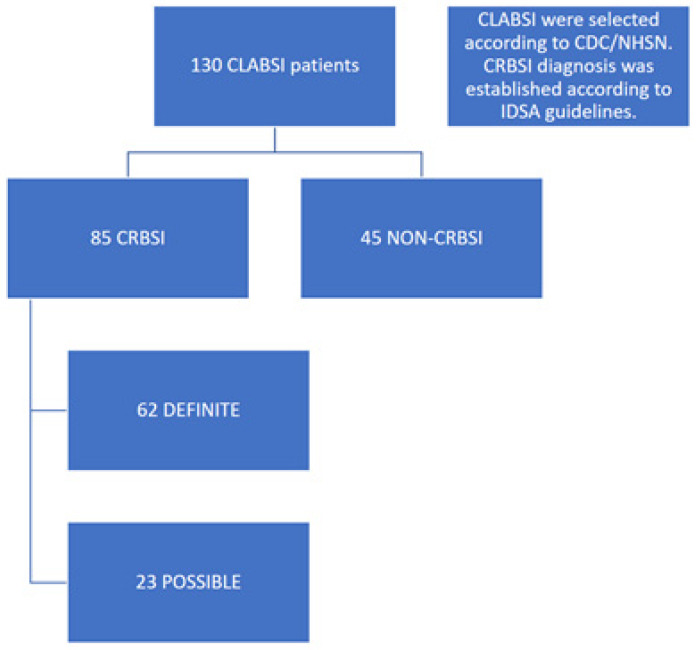
Flowchart for patients recruited into the study.

The inclusion criteria were patients aged 18 years and above, diagnosed with end-stage kidney disease and receiving maintenance HD with a catheter, and having a CVC in situ for at least 7 days. Patients in whom a source of infection other than the catheter (such as pneumonia and urinary tract infection) were excluded from the study. From hospital records, 130 patients were enrolled using the CDC/NHSN definition of CLABSI. Data were collected. This group was further detailed according to the IDSA definition of CRBSI. Only 85 patients met the IDSA definition of CRBSI criteria for the analytic cohort. The other patients were excluded from the study (Figure 1). Data were extracted from the electronic health record system by designated investigators. To ensure patient confidentiality, all personal identifiers (e.g., citizen ID numbers and contact information) were removed at the point of extraction. The final dataset was stored on a password-protected, encrypted server, accessible only to the primary research team, in full compliance with the Declaration of Helsinki. The study protocol received official approval from the local ethics committee (Antalya Akdeniz University Hospital Local Ethics Committee; 2023-336, 26 April 2023).

Institutions implement standardized bundles categorized into insertion and maintenance phases for preventing and managing CLABSI and CRBSI. The insertion bundle must prioritize aseptic techniques, requiring rigorous hand hygiene, maximal sterile barrier precautions, and the use of alcoholic chlorhexidine for skin antisepsis. The maintenance phase relies on daily assessments to remove unnecessary lines and the practice of “scrubbing the hub” with antiseptics before every access to prevent intraluminal contamination [18]. Even without clear cardiac symptoms, transthoracic echocardiography is often indicated if blood cultures are positive for *Staphylococcus aureus*, as this pathogen has a high propensity for endocardial seeding [16,19].

Regarding management, institutional catheter removal policies should mandate the immediate extraction of the device if the infection involves virulent pathogens, such as *Staphylococcus aureus* or Candida, *Pseudomonas aeruginosa*, or Mycobacteria or severe clinical status (signs of severe sepsis, septic shock, or hemodynamic instability) or if there is evidence of IE or suppurative thrombophlebitis, or osteomyelitis or other metastatic infections and persistent bacteremia [16].

### 2.4. Microbiological Methods

Simultaneous blood cultures from the catheter hub and a peripheral vein were obtained, followed by the immediate administration of empirical antibiotics. At least two sets of blood cultures (for aerobic and anaerobic incubation) were obtained at the disease onset whenever possible. Organism identification and antimicrobial susceptibility patterns were determined using standardized automated methods. Septic pulmonary emboli were diagnosed using computed tomography of the lungs.

### 2.5. Statistical Analyses

All the collected data was entered into the Statistical Package for the Social Sciences version 22 software for further data cleaning and statistical analysis. Descriptive statistics were used to summarize the demographic and clinical characteristics. Continuous variables are reported as mean ± standard deviation for normally distributed data or median (interquartile range Q1–Q3) for skewed data. Categorical variables are reported as numbers (percentages). Comparisons between the groups were made using the chi-square test for categorical data. For continuous data, normality was assessed using the Shapiro–Wilk test. For non-normally distributed continuous variables, the Mann–Whitney U test was applied. Multivariable binary logistic regression analysis was performed to identify independent predictors of 90-day mortality.

## 3. Results

### 3.1. Demographic Data and Baseline Characteristics

The final analytic cohort consisted of 85 patients. The median age was 56 (42.5–67) years. The majority were men (63.5%), and common comorbidities included hypertension (64.7%) and diabetes mellitus (40%). Hypertensive kidney disease was the main etiology of end-stage kidney disease (31.8%). 19 patients (22.4%) were on immunosuppressants and returned to dialysis after graft failure. Five patients (5.9%) had malignancies. Table 1 provides a comprehensive summary of the patients’ characteristics and laboratory results.

A total of 72.9% of patients had a tunneled catheter. The current locations of all catheters (tunneled and non-tunneled) were as follows: right jugular 43 (50.6%), left jugular 8 (9.4%), right femoral 11 (12.9%), left femoral 9 (10.6%), right subclavian 10 (11.8%), left subclavian 4 (4.7%).

CRBSI was classified as definite in 62 patients and possible in 23 according to the IDSA definition. Notably, IE was diagnosed in 9.4% (n:8) of the catheter-dependent HD patients with CRBSI according to Duke-ISCVID 2023 criteria. Five patients were diagnosed with definite IE (2 major criteria), two patients with possible IE (one major + one minor) criteria, and one patient with possible IE (three minor criteria). The most frequent pathogen in the IE group was *Staphylococcus aureus* (5/8 patients, 62.5%). Two patients underwent valve surgery, and one underwent surgery due to frequent blood clots in the brain. The second patient (in the non-IE group), a kidney transplant candidate, required surgical intervention after an organized right atrial thrombus was identified as the final diagnosis. In the IE group, three patients presented with septic pulmonary embolism, and one patient presented with a cerebrovascular embolism (Table 1).

### 3.2. Comparison Between Non-IE and IE Patients

A clear statistical difference was found between the IE and non-IE groups with respect to vegetation, embolism, length of hospitalization (*p* < 0.05) (Table 1). Moreover, 16 patients did not have echocardiographic results. Five patients had vegetation on the mitral valve, while two patients had vegetation on the tricuspid valve. A thrombus was detected at the catheter tip in five patients who did not have infective endocarditis.

### 3.3. Microbiology Profiles

Only one episode of catheter infection was recorded per patient. Table 2 shows the etiology of catheter-related bacteremia from catheter hub culture. Of the pathogens isolated from catheter cultures, Gram-positive organisms constituted 70.6% of cases. Among these, *Staphylococcus aureus* was the leading cause of all 85 CRBSI patients, isolated in 30 patients (35.3%). MRSA growth was observed in five patients, and IE was detected in two of them. Gram-negative bacteria accounted for 29.4% of all catheter cultures. In particular, nearly all Gram-positive bacterial isolates in the IE group were *Staphylococcus aureus* (5/6). Apart from *Staphylococcus aureus*, blood cultures from the patient with IE yielded *Stenotrophomonas maltophilia* (n:1), *Enterobacter cloacae* (n:1), and *Enterococcus faecalis* (n:1). Although there was bacterial growth in the catheter hub, there was no bacterial growth in the blood cultures of 12 (14.1%) patients. All non-tunneled catheters and all catheters growing MRSA were removed. Catheters yielding growth of *Enterobacter cloacae* (*n* = 2), *Klebsiella pneumoniae* (*n* = 1), *Enterobacter aerogenes* (*n* = 1), *Coagulase-negative staphylococci* (*n* = 2), and *Enterococcus faecalis* (*n* = 2) were not removed, whereas all other catheters were removed.

### 3.4. Mortality

In-hospital mortality was 5.2% (4/77) for the non-IE group and 12.5% (1/8) for the IE group. Moreover, 7 of 85 patients (8.2%) died within 28 days of admission, and 12 of 85 patients (14.1%) died within 90 days of admission (Table 1). The 90-day all-cause mortality was similar in both groups. Of those who died, one had severe heart failure prior to hospitalization, one had undergone mitral valve replacement before hospitalization fifteen years ago, and one had atrial fibrillation.

Clinical characteristics and laboratory findings of patients who survived and those who died within 90 days are compared in Table 3.

The echocardiogram results for 16 patients were missing. A total of 5 of them were in the deceased group, and 11 were in survived group. Mitral A wave (*p* = 0.058) showed a trend towards significance, suggesting they may have clinical relevance despite not reaching the 0.05 threshold in this sample size. The remaining echocardiographic findings did not reach statistical significance in relation to 90-day mortality (Table 4).

In the univariable logistic regression for 28-day all-cause mortality, age was the only variable identified as a statistically significant predictor (OR: 1.070; 95% CI: 1.007–1.137; *p* = 0.029) (See Supplementary Material Table S3). Similarly, in the univariable analysis for 90-day all-cause mortality, both age and female gender emerged as significant predictors (*p* < 0.05). However, multivariable binary logistic regression confirmed that only age (OR: 1.055; 95% CI: 1.005–1.107; *p* = 0.029) remained an independent significant predictor of 90-day all-cause mortality within the total CRBSI cohort (*n* = 85) (Table 5).

## 4. Discussion

To the best of our knowledge, this is the first study to have examined only the IE incidence rate and 90-day all-cause mortality for hospitalized catheter-dependent HD patients with CRBSI. The 90-day all-cause mortality was 14.1% (12/85). Although univariate analysis identified that older age and female gender were associated with increased mortality, in the multivariable binary logistic regression analysis, only age remained an independent factor significantly associated with 90-day all-cause mortality.

The high prevalence of tunneled catheters (72.9%) in our cohort aligns with trends reported in other regions, including United States Renal Data System (USRDS) and the REIN register in France, and in Turkey, respectively, 72.2%, 56.7% and 68.7% of patients initiate HD, where CVCs unfortunately remain the predominant mode of vascular access for initiating renal replacement therapy due to the insufficient arteriovenous fistula formation before renal replacement therapy [20,21,22]. This high catheter usage undoubtedly contributes to the elevated risk and subsequent cost associated with CRBSI and its complications.

Gram-positive organisms predominated in our cohort, accounting for 70.6% of catheter hub isolates. Specifically, *Staphylococcus aureus* was identified in 35.3% (30/85) of all catheter hub cultures, representing the leading cause of infection. Gram-negative bacteria were isolated in 29.4% of all CRBSI cases. The microbial distribution in our study population aligns with previously published data. Gram-positive aerobes remain the predominant pathogens in CRBSIs, with *Staphylococcus aureus* and *Coagulase-negative staphylococci* accounting for 60–80% of cases involving tunneled catheter [23,24]. Conversely, Gram-negative bacilli contribute to roughly 20% of CRBSIs [25].

CRBSI in HD patients, particularly those caused by *Staphylococcus aureus*, have been associated with a higher risk for hematogenous complications [21,26]. In our study, nearly all cases of endocarditis with Gram-positive bacterial isolates were caused by *Staphylococcus aureus* (5/8) and were associated with a higher rate of hematogenous complications (3 out of 5 patients). The sole Gram-negative case presenting with septic pulmonary emboli was caused by *Stenotrophomonas maltophilia*. In one study, Farrington et al. found polymicrobial infections in 40 (14%) of 289 patients with CRBSI [4]. In our study population, dual microorganism growth was detected in two (2.4%) patients, who showed a combination of Gram-negative growths; both of them have malignancy.

Data regarding vascular access sites confirm that catheters placed in the internal jugular vein carry a reduced probability of infection. This pattern aligns with existing studies, which have demonstrated that the femoral site is associated with a greater likelihood of bacteremia than either the subclavian or internal jugular insertion points [23,24,25,27]. Although in our study the internal jugular vein was the main site of placement, we still encountered CRBSI.

Patients undergoing HD experience a significantly magnified risk of IE, with an incidence rate of 1.7–2.0 cases per 1000 patients. This figure highlights a disparity of over 50-fold compared to the risk level found in those without end-stage renal disease [13]. A meta-analysis including 45,799 patients with IE on HD showed that approximately 2.7–3.1% of the HD group suffer from endocarditis [13]. The higher incidence of IE observed in our study compared to previous reports can be attributed to our specific inclusion criteria, which focused exclusively on patients hospitalized with CRBSIs. In contrast to other studies where catheter use ranged from 26.4% to 79.4 [14,28,29,30], our cohort consisted of hospitalized catheter-dependent HD patients, a population inherently at the highest risk for developing IE. Data from a 2024 retrospective study show that among a cohort of 254 CRBSI patients, the HD-specific subgroup (*n* = 12) exhibited an infective endocarditis rate of 8.3% [25].

A meta-analysis found an overall in-hospital death rate of 29.5% (95% CI: 26.7–46.6%) IE in the context of HD [13]. Specifically, the one-year survival prognosis for HD patients affected by IE in the United States is reported to be 46% [31]. In our study, one of the eight IE patients died during hospitalization; none of the remaining patients died within 90 days. Our study did not yield a statistically significant difference in survival outcomes between the IE and non-IE groups; this lack of significance may be attributed to the limited sample size.

Some investigators found that females predominated in IE cases among HD patients, whereas other investigators noted a majority of males [28,29,30,32]. This disparity is potentially attributable to geographical variations among the investigated cohorts. In our study, there was no difference between sexes for IE, but female patients had a higher mortality rate in CRBSI cases.

The optimal management strategy for IE in HD patients remains a subject of debate. A recent meta-analysis by Ting et al. confirmed that survival outcomes do not significantly differ between surgical and medical interventions in this high-risk population [33]. Furthermore, the prognosis remains particularly grim for those requiring surgery; Elderia et al. recently reported a 1-year mortality rate as high as 75.6% [34]. One of our patients survived.

In light of the high prevalence of *Staphylococcus aureus* reported both in our cohort and by Stahl et al. (58%) [35], clinicians should maintain a high index of suspicion for IE, particularly when this pathogen is isolated [16]. Furthermore, in some studies, researchers reported negative blood cultures in nearly half of their study IE in HD patients population [5,30,32]. Wang et al. reported positive culture rates exceeding 70% in their investigations [2]. One of the major criteria for IE is the growth of microorganisms. If there is no growth, an echocardiography is required for the other major criterion as well. Some of the minor criteria also have an echocardiography finding. In echocardiography, Mitral A wave velocity reflects the Left Atrium–Left Ventricle pressure gradient during late diastole, which is affected by Left Ventricle compliance and Left Atrium contractile function. Mitral A wave velocity increases with aging. Furthermore, normal aging is associated with a number of changes in the heart and vascular system, especially slowing of LV relaxation which may lead to diastolic dysfunction [36]. While aging is typically associated with a compensatory increase in A wave velocity (impaired relaxation), our findings suggest that a low Mitral A wave in this cohort might serves as a marker of advanced restrictive pathology. In our study, although not reaching statistical significance, the lower Mitral A wave velocity in the mortality group (0.6 vs. 0.9 m/s, *p* = 0.058) might suggests a potential impairment in atrial contraction and diastolic filling dynamics. Patients without echocardiographic evaluation had a median hospitalization of 14.5 days (Q1–Q3 = 13–20.7), which provided an adequate window for clinical monitoring. The absence of late-emerging symptoms or clinical deterioration during this period suggests that the lack of imaging in these individuals did not lead to an underestimation of the IE incidence within our CRBSI cohort. Echocardiography also enable the detection of thrombi at the catheter tip. Given that IE can occur even in the presence of negative culture [14,35], and is associated with high mortality rates regardless of surgical intervention, echocardiography might be considered a standard diagnostic procedure for hospitalized catheter dependent HD patients presenting with CRBSI.

As the high rates of hospitalization and morbidity associated with CLABSI, prevention is the primary clinical objective. To achieve this, standardized dialysis event prevention bundles have been developed as the gold standard of care. Dialysis event prevention bundles encompass various components, such as infection control measurements in hemodialysis catheter connection, hemodialysis catheter disconnection, hemodialysis catheter exit site care, dialysis station routine disinfection, hemodialysis injectable medication preparation, hemodialysis injectable medication administration and hand hygiene [37].

Several methodological limitations should be considered when interpreting these results. Foremost, the reliance on a retrospective, observational design utilizing registry data inherently precludes the inference of definitive causal relationships. While the current study may be underpowered to detect subtle survival differences, further investigation in larger cohorts would remedy this issue. Since this study includes patients from both our hospital’s HD unit and various external hemodialysis centers, full adherence to the prevention bundle protocols could not be independently verified for all cases. We were also unable to determine the specific incidence rates of CRBSI and IE exclusive to our own HD unit.

## 5. Conclusions

This 7-year study highlights that CRBSI remains a major infectious complication in catheter-dependent HD patients, with *Staphylococcus aureus* as the predominant pathogen in both CRBSI and IE. Within our cohort, higher age was found to be an independent predictor of 90-day all-cause mortality, though the small sample size remains a limitation. Our study identifies a notable 9.4% (8/85) incidence of IE among catheter-dependent HD patients hospitalized with CRBSI. Preventive efforts should focus on modifiable risk factors, particularly stringent infection control practices, reducing Gram-positive growth, standardized catheter care, and early transition to arteriovenous fistulas. Future large-scale multicenter studies are warranted to further elucidate survival predictors for hospitalized hemodialysis patients complicated by CRBSI and IE.

## Figures and Tables

**Table 1:** Comparison of infective endocarditis and non-infective endocarditis patients.

	Total (*n* = 85)	Non-Infective Endocarditis (*n* = 77)	Infective Endocarditis (*n* = 8)	*p*
Age in years	56 (42.5–67)	56 (41.5–67)	56.5 (49.5–69.8)	0.760
Sex (Female/Male) (female%)	31/54 (36.5%)	27/50 (35.1%)	4/4 (50.0%)	0.455
CKD etiology				
- Hypertension	27 (31.8%)	25 (32.5%)	2 (25.0%)	
- Diabetes mellitus	23 (27.1%)	20 (26.0%)	3 (37.5%)	
- Unknown	13 (15.3%)	11 (14.3%)	2 (25.0%)	
- Others	8 (9.4%)	8 (10.4%)	0 (0.0%)	0.836
- Obstructive reasons	7 (8.2%)	6 (7.8%)	1 (12.5%)	
- Chronic glomerulonephritis	6 (7.1%)	6 (7.8%)	0 (0.0%)	
- ADPKD	1 (1.2%)	1 (1.3%)	0 (0.0%)	
Diabetes Mellitus (yes %)	34 (40%)	31 (40.3%)	3 (37.5%)	1.000
Hypertension (yes %)	55 (64.7%)	50 (64.9%)	5 (62.5%)	1.000
Heart Failure history (yes %)	8 (9.4%)	8 (10.4%)	0 (0.0%)	1.000
Malignancy (yes %)	5 (5.9%)	4 (5.2%)	1 (12.5%)	0.398
Renal transplantation (yes %)	19 (22.4%)	17 (22.1%)	2 (25.0%)	1.000
Tunneled Catheter (%)	62 (72.9%)	55 (71.4%)	7 (87.5%)	0.439
Catheter time (days)	30.0 (14.9–247.5)	30.0 (14.4–240)	90.0 (21–540)	0.440
Vegetation (yes %) (n:69)	7 (10.1%)	0 (0.0%)	7 (87.5%)	<0.001
Embolism (yes %)	4 (4.7%)	0 (0.0%)	4 (50.0%)	<0.001
Culture of catheter				
- G-positive	60 (70.6%)	54 (70.1%)	6 (75%)	1.000
- G-negative	25 (29.4%)	23 (29.9%)	2 (25%)	
Need for surgery (yes %)	2 (2.4%)	1 (1.3%)	1 (12.5%)	0.180
Mortality in hospital, (yes%)	5 (5.9%)	4 (5.2%)	1 (12.5%)	0.390
Mortality in 28 days, (yes%)	7 (8.2%)	6 (7.8%)	1 (12.5%)	0.500
Mortality in 90 days, (yes%)	12 (14.1%)	11 (14.3%)	1 (12.5%)	1.000
Catheter Removal (yes%)	62 (72.9%)	54 (70.1%)	8 (100%)	0.100
Hospitalization days	18 (14–30)	17 (14–24)	48 (42–73.8)	<0.001
WBC (/mL)	10,120 (6365–13,385)	9890 (6365–13,260)	12,330 (5757–16,480)	0.450
Neutrophil (/mL)	7200 (4505–11,180)	6800 (4505–11,070)	10,165 (3542–14,440)	0.380
Lymphocyte (/mL)	780 (480–1490)	740 (460–1515)	940 (555–1442)	0.800
Hemoglobin (g/dL)	9.2 (8.4–10.4)	9.3 (8.55–10.4)	8.4 (8.15–9.05)	0.085
Platelet (×10^3^/mL)	180 (130–242)	182 (127.5–242)	166 (148–256)	0.880
CRP (mg/L)	120.5 (69.6–173)	119.0 (64–164.6)	150.5 (94.8–194.9)	0.210
Albumin (g/dL)	3.4 (3.1–3.6)	3.4 (3.16–3.6)	3.2 (2.9–3.5)	0.190

Data are presented as median (Q1–Q3). CKD: Chronic Kidney Disease, ADPKD: Autosomal Dominant Polycystic Kidney Disease.

**Table 2:** Distribution of microbial isolates of catheter-related bacteremia based on catheter hub culture.

		Non-Infective Endocarditis (*n* = 77)	Infective Endocarditis (*n* = 8)
Gram-positive	*Staphylococcus aureus*	25 (32.5%)	5 (62.5%)
	Coagulase-negative staphylococci	10 (13%)	
	*Enterococcus faecalis*	6 (7.8%)	1 (12.5%)
	*Staphylococcus epidermidis*	4 (5.2%)	
	*Staphylococcus hominis*	2 (2.6%)	
	*Streptococus mitis*	1 (1.3%)	
	*Kocuria rhizophila*	1 (1.3%)	
	Diphtheroid basil	1 (1.3%)	
	*Micrococcus luteus*	1 (1.3%)	
	*Streptecocus oralis*	1 (1.3%)	
	*Enterococcus faecium*	1 (1.3%)	
	*Enterococcus hirae*	1 (1.3%)	
	Total	54 (70.1%)	6 (75%)
Gram-negative	*Klebsiella pneumoniae*	5 (6.5%)	
	*Enterobacter cloacae*	5 (6.5%)	1 (12.5%)
	*Klebsiella oxytoca*	2 (2.6%)	
	*Pseudomonas stutzeri*	2 (2.6%)	
	*Enterobacter asburiae*	2 (2.6%)	
	*Escherichia coli*	1 (1.3%)	
	*Rhizobium radiobacter*	1 (1.3%)	
	*Stenotrophomonas maltophilia*		1 (12.5%)
	*Moraxella osloensis*	1 (1.3%)	
	*Pseudomonas aeruginosa*	1 (1.3%)	
	*Pantoea agglomerans*	1 (1.3%)	
	Enterobacteriaceae spp.	1 (1.3%)	
	*Enterobacter aerogenes*	1 (1.3%)	
	Total	23 (29.9%)	2 (25%)

**Table 3:** Comparison of patients who survived and died within 90 days.

	Total (*n* = 85)	Survived (*n* = 73)	Died (*n* = 12)	*p*
Age in years	56 (42.5–67)	55 (41–65)	67.0 (53.75–75.75)	0.012
Sex Female/Male, (Female%)	31/54 (36.5%)	23/50 (31.5%)	8/4 (66.7%)	0.026
CKD etiology				
- Hypertension	27 (31.8%)	23 (31.5%)	4 (33.3%)	
- Diabetes mellitus	23 (27.1%)	21 (28.8%)	2 (16.7%)	
- Unknown	13 (15.3%)	9 (12.3%)	4 (33.3%)	0.345
- Others	8 (9.4%)	6 (8.2%)	2 (16.7%)	
- Obstructive reasons	7 (8.2%)	7 (9.6%)	0	
- Chronic glomerulonephritis	6 (7.1%)	6 (8.2%)	0	
- ADPKD	1 (1.2%)	1 (1.4%)	0	
Diabetes Mellitus (yes%)	34 (40.0%)	31 (42.5%)	3 (25.0%)	0.347
Hypertension (yes%)	55 (64.7%)	50 (68.5%)	5 (41.7%)	0.103
Heart failure history (yes%)	8 (9.4%)	5 (6.8%)	3 (25.0%)	0.081
Tunneled catheter (yes%)	62 (72.9%)	54 (74%)	8 (66.7%)	0.700
Renal transplantation (yes%)	19 (22.4%)	17 (23.3%)	2 (16.7%)	1.000
Catheter time, days	30 (14.9–247.5)	30 (15–202.5)	150.0 (23.8–540)	0.287
Malignancy (yes%)	5 (5.9%)	4 (5.5%)	1 (8.3%)	0.542
Embolism (yes%)	4 (4.7%)	4 (5.5%)	0 (0.0%)	1.000
Need for surgery (yes%)	2 (2.4%)	2 (2.7%)	0 (0.0%)	1.000
Catheter Removal (yes %)	62 (72.9%)	54 (74%)	8 (66.7%)	0.700
Hospitalization days	18 (14–30)	18.0 (14–27)	21.0 (15.3–39.8)	0.362
Culture of catheter				
- G-positive	60 (70.6%)	51 (69.9%)	9 (75%)	1.000
- G-negative	25 (29.4%)	22 (30.1%)	3 (25%)	
White blood cells (/mL)	10,120 (6365–13,385)	9550 (6290–13,385)	10,685 (9425–14,985)	0.453
Neutrophil (/mL)	7200 (4505–11,180)	6630 (4205–11,070)	9500 (6840–13,610)	0.238
Lymphocyte (/mL)	780 (480–1490)	790 (480–1490)	720 (432–1530)	0.738
Hemoglobin (g/dL)	9.2 (8.4–10.4)	9.1 (8.15–10.4)	9.6 (9.025–10.4)	0.309
Platelet (×10^3^/mL)	180 (130–242)	180 (130–240)	194.5 (94.5–298.8)	0.714
CRP (mg/L)	120.5 (69.6–173)	115.9 (63.95–168.5)	138.1 (99.75–179.3)	0.354
Albumin (g/dL)	3.4 (3.1–3.6)	3.4 (3.1–3.67)	3.2 (2.8–3.5)	0.110

Data are presented as median (Q1–Q3). CKD: Chronic Kidney Disease, ADPKD: Autosomal Dominant Polycystic Kidney Disease.

**Table 4:** Echocardiographic results in survivors and those who died within 90 days: Analysis of patients with available results.

	Total (*n* = 69)	Survived (*n* = 62)	Died (*n* = 7)	*p*
Vegetation (yes%)	7 (10.1%)	6 (9.7%)	1 (14.3%)	0.544
Ejection fraction %	63 (56–65)	64 (55–65)	60 (59–65)	0.683
Aortic root (cm)	2.8 (2.8–3.1)	2.9 (2.7–3.1)	2.8 (2.8–3.2)	0.687
Left atrium (cm)	3.9 (3.5–4.3)	3.9 (3.5–4.2)	4.4 (3.5–4.5)	0.213
LV end-diastolic diam. (cm)	4.6 (4.2–5)	4.6 (4.2–5.025)	4.5 (4.4–4.8)	0.834
LV end-systolic diam. (cm)	3 (2.7–3.4)	3 (2.7–3.4)	2.9 (2.7–3.5)	0.968
Interventricular septum (cm)	1.2 (1.1–1.35)	1.2 (1.1–1.325)	1.2 (1.1–1.4)	0.770
LV posterior wall (cm)	1.2 (1.1–1.3)	1.2 (1.1–1.3)	1.2 (1.1–1.3)	0.864
Mitral E wave (m/s)	0.9 (0.6–1.2)	0.9 (0.6–1.2)	0.9 (0.8–1.3)	0.582
Mitral A wave (m/s)	0.9 (0.7–1.0)	0.9 (0.7–1.0)	0.6 (0.5–1)	0.058
Aorta velocity (m/s)	1.5 (1.3–1.8)	1.5 (1.3–1.9)	1.3 (1.2–1.7)	0.127
Aortic apex gradient (mmHg)	9.0 (6.8–12.7)	9.0 (6.8–13.7)	8 (6.8–11.6)	0.522
TR max velocity (m/s)	2.5 (2.3–2.9)	2.5 (2.3–2.8)	2.9 (2.3–3.1)	0.490
Pulmonary velocity (m/s)	1 (0.9–1.1)	0.9 (1–1.1)	0.9 (0.8–1.2)	0.890
Segmental movement defect (yes%)	14 (20.6%)	12 (19.7%)	2 (28.6%)	0.627
Pericardial effusion (yes%)	18 (26.1%)	17 (27.4%)	1 (14.3%)	0.660
Pulmonary pressure (mmHg)	37 (33–45)	37 (33–43.5)	45 (32–47.5)	0.601

LV: left ventricle, TR: Tricuspid Regurgitation.

**Table 5:** Multivariable binary logistic regression analysis for 90-day all-cause mortality.

Variable	Univariable Analysis OR (95% CI)	*p*	Multivariable Analysis OR (95% CI)	*p*
Age	1.060 (1.012–1.11)	0.014	1.055, 1.005–1.107	0.029
Sex (Female)	4.34 (1.187–15.919)	0.026		

CI: Confidence interval.

## Data Availability

Data is unavailable due to privacy or ethical restrictions; a statement is still required.

## Supplementary Materials

The following supporting information can be downloaded at: https://www.mdpi.com/article/10.3390/jcm15051815/s1, Table S1: Comparison of baseline characteristics and laboratory findings between survivors and non-survivors at 28 days, Table S2: Available echo-cardiographic results in survivors and non-survivors at 28 days, Table S3: Univariable binary logistic regression analysis for 28-day all- cause mortality.

## Abbreviations

The following abbreviations are used in this manuscript:

CI	Confidence interval
CKD	Chronic kidney disease
CRBSI	Catheter-related bloodstream infections
CVC	Central venous catheter
HD	Hemodialysis
IE	Infective endocarditis
ADPKD	Autosomal Dominant Polycystic Kidney Disease
LV	Left Ventricle
TR	Tricuspid Regurgitation

## Appendix A

### Appendix A.1. Definition of Catheter-Related Bloodstream Infections (CRBSI) [16]

A definitive diagnosis of CRBSI requires that the same organism grow from at least one percutaneous blood culture and from a culture of the catheter tip (A-I), or that two blood samples be drawn (one from a catheter hub and the other from a peripheral vein) that, when cultured, meet CRBSI criteria for quantitative blood cultures or differential time to positivity (DTP) (A-II). Alternatively, two quantitative blood cultures of samples obtained through two catheter lumens in which the colony count for the blood sample drawn through one lumen is at least three-fold greater than the colony count for the blood sample obtained from the second lumen should be considered to indicate possible CRBSI (B-II).

When a peripheral blood sample cannot be obtained, no other catheter is in place from which to obtain an additional blood sample, there is no drainage from the insertion site available for culture, and there is no clinical evidence for an alternate source of infection, then positive results of culture performed on a blood sample obtained from a catheter should lead to continuation of antimicrobial therapy for possible CRBSI in a symptomatic hemodialysis patient (B-II).

### Appendix A.2. Definitions of Terms Used in the 2023 Duke-International Society for Cardiovascular Infectious Diseases Infective Endocarditis (IE) Criteria for the Diagnosis of IE [17]

I. MAJOR CRITERIA

A. Microbiologic Major Criteria

(1) Positive blood cultures

i. Microorganisms that commonly cause IE isolated from two or more separate blood culture sets (Typical)

or

ii. Microorganisms that occasionally or rarely cause IE isolated from three or more separate blood culture sets (Nontypical)

(2) Positive laboratory tests

i. Positive polymerase chain reaction (PCR) or other nucleic acid-based technique for *Coxiella burnetii*, Bartonella species, or *Tropheryma whipplei* from blood

or

ii. *Coxiella burnetii* antiphase I immunoglobulin G (IgG) antibody titer > 1:800, or isolated from a single blood culture

or

iii. Indirect immunofluorescence assays (IFA) for detection of IgM and IgG antibodies to *Bartonella henselae* or *Bartonella quintana* with immunoglobulin G (IgG) titer ≥ 1:800

B. Imaging Major Criteria

(1) Echocardiography and cardiac computed tomography (CT) imaging

i. Echocardiography and/or cardiac CT showing vegetation, valvular/leaflet perforation, valvular/leaflet aneurysm, abscess, pseudoaneurysm, or intracardiac fistula

or

ii. Significant new valvular regurgitation on echocardiography as compared with previous imaging. Worsening or changing of pre-existing regurgitation is not sufficient.

or

iii. New partial dehiscence of prosthetic valve as compared with previous imaging

(2) Positron emission computed tomography with 18F-fluorodeoxyglucose ([18F] FDG PET/CT imaging)

Abnormal metabolic activity involving a native or prosthetic valve, ascending aortic graft (with concomitant evidence of valve involvement), intracardiac device leads or other prosthetic material.

C. Surgical Major Criteria

Evidence of IE documented by direct inspection during heart surgery, neither Major Imaging Criteria nor subsequent histologic or microbiologic confirmation.

II. MINOR CRITERIA

A. Predisposition

- Previous history of IE
- Prosthetic valve
- Previous valve repair
- Congenital heart disease
- More than mild regurgitation or stenosis of any etiology
- Endovascular intracardiac implantable electronic device (CIED)
- Hypertrophic obstructive cardiomyopathy
- Injection drug use

B. Fever: documented temperature greater than 38.0 °C (100.4 °F)

C. Vascular phenomena: clinical or radiological evidence of arterial emboli, septic pulmonary infarcts, cerebral or splenic abscess, mycotic aneurysm, intracranial hemorrhage, conjunctival hemorrhages, Janeway lesions, purulent purpura

D. Immunologic Phenomena Positive rheumatoid factor, Osler nodes, Roth spots, or immune complex-mediated glomerulonephritis

E. Microbiologic Evidence, Falling Short of a Major Criterion

(1) Positive blood cultures for a microorganism consistent with IE, but not meeting the requirements for Major Criterion

or

(2) Positive culture, PCR, or other nucleic acid-based test (amplicon or shotgun sequencing, in situ hybridization) for an organism consistent with IE from a sterile body site other than cardiac tissue, cardiac prosthesis, or arterial embolus; or a single finding of a skin bacterium by PCR on a valve or wire without additional clinical or microbiological supporting evidence

F. Imaging Criteria

Abnormal metabolic activity as detected by [18F] FDG PET/CT within 3 mo. of implantation of prosthetic valve, ascending aortic graft (with concomitant evidence of valve involvement), intracardiac device leads or other prosthetic material.

G. Physical Examination Criteria’s

New valvular regurgitation identified on auscultation if echocardiography is not available. Worsening or changing of pre-existing murmur is not sufficient.

### Appendix A.3. Definitions of Terms Used in the 2023 Duke-International Society for Cardiovascular Infectious Diseases Infective Endocarditis (IE) Criteria for the Diagnosis of IE

I. **DEFINITE ENDOCARDITIS**

A. Pathologic Criteria

(1) Microorganisms identified in the context of clinical signs of active endocarditis in a vegetation; from cardiac tissue; from an explanted prosthetic valve or sewing ring; from an ascending aortic graft (with concomitant evidence of valve involvement); from an endovascular intracardiac implantable electronic device (CIED); or from an arterial embolus

or

(2) Active endocarditis (may be acute or subacute/chronic identified in or on a vegetation; from cardiac tissue; from an explanted prosthetic valve or sewing ring; from an ascending aortic graft (with concomitant evidence of valve involvement); from a CIED; or from an arterial embolus

B. Clinical Criteria

(1) Two Major Criteria

or

(2) One Major Criterion and Three Minor Criteria

or

(3) Five Minor Criteria

II. **POSSIBLE ENDOCARDITIS**

A. 1 Major Criterion and 1 Minor Criterion

or

B. Three Minor Criteria

III. **REJECTED ENDOCARDITIS**

A. Firm alternate diagnosis explaining signs/symptoms

or

B. Lack of recurrence despite antibiotic therapy for less than 4 d.

or

C. No pathologic or macroscopic evidence of IE at surgery or autopsy, with antibiotic therapy for less than 4 d

or

D. Does not meet criteria for possible IE, as above.
