# Supplementary material for: Microorganisms and Mortality Factors in Hospitalized Hemodialysis Patients with Catheter-Related Bloodstream Infection and Infective Endocarditis: 7 Years of Experience
Source: J Clin Med. 2026 Feb 27;15(5):1815. doi: 10.3390/jcm15051815 (PMC12985569; doi:10.3390/jcm15051815)
Supplement: Supplementary file 1 [file jcm-15-01815-s001.zip › jcm-4117530-supplementary.pdf]

Table S1: Comparison of baseline characteristics and laboratory findings between survivors and non-survivors at 28 days

	Total (n=85)	Survived (n=78)	Died (n=7)	p-value
Age in years	56(42.5-67)	55.5 (41.0-66)	67.0 (52-86.0)	0.035
Sex Female/Male, (Female%)	31/54 (36.5%)	28/50 (35.9%)	3/4 (42.9%)	0.702
CKD etiology				
- Hypertension	27 (31.8%)	25 (32.1%)	2 (28.6%)	
- Diabetes mellitus	23 (27.1%)	22 (28.2%)	1 (14.3%)	
- Unknown	13 (15.3%)	10 (12.8%)	3 (42.9%)	
- Others	8 (9.4%)	7 (9.0%)	1 (14.3%)	0.446
- Obstructive reasons	7 (8.2%)	7 (9.0%)	0 (0.0%)	
-Chronic glomerulonephritis	6 (7.1%)	6 (7.7%)	0 (0.0%)	
- ADPKD	1 (1.2%)	1 (1.3%)	0 (0.0%)	
Diabetes Mellitus (yes%)	34 (40.0%)	33 (42.3%)	1 (14.3%)	0.234
Hypertension (yes%)	55 (64.7%)	53 (67.9%)	2 (28.6%)	0.091
Heart failure history (yes%)	8 (9.4%)	7 (9.0%)	1 (14.3%)	0.513
Tunneled catheter (yes%)	62 (72.9%)	4 (5.1%)	1 (14.3%)	0.356
Renal transplantation (yes%)	19 (22.4%)	18 (23.1%)	1 (14.3%)	1.000
Catheter time, days	30 (14.9-247.5)	59 (75.6%)	3 (42.9%)	0.082
Malignancy (yes%)	5 (5.9%)	30.0 (15.0-210.0)	120.0 (5-540.0)	0.459
Embolism (yes%)	4 (4.7%)	57 (73.1%)	5 (71.4%)	1.000
Need for surgery (yes%)	2 (2.4%)	4 (5.1%)	0 (0.0%)	1.000
Catheter Removal (yes %)	62 (72.9%)	2 (2.6%)	0 (0.0%)	1.000
Hospitalization days	18 (14-30)	18.0 (14.0-30)	16.0 (9-42)	0.580
Culture of catheter				
- G-positive	60 (70.6%)	56 (71.8%)	4 (57.1%)	0.414
- G-negative	25 (29.4%)	22 (28.2%)	3 (42.9%)	0.414
White blood cells (/mL)	10120(6365-13385)	10190 (6382-13675)	10091 (5990-13200)	0.955
Neutrophil (/mL)	7200 (4505-11180)	7000 (4505-11170)	9810 (4510-11570)	0.707
Lymphocyte (/mL)	780(480-1490)	795 (490-1532)	660 (350-1010)	0.419
Hemoglobin (g/dL)	9.2 (8.4-10.4)	9.15 (8.3-10.4)	9.2 (9.0-10.0)	0.725
Platelet ($X10^3$/mL)	180 (130-242)	181 (128-241)	167 (159-253)	0.817
CRP (mg/L)	120.5 (69.6-173)	117.8 (65.5-167.3)	161.6 (94.1-195.5)	0.201
Albumin (g/dL)	3.4 (3.1-3.6)	3.4 (3.1-3.6)	3.2 (2.2-3.5)	0.116

Table S2: Available echocardiographic results in survivors and non-survivors at 28 days

	**Total (n=69)**	**Survived (n=65)**	**Died (n=4)**	**p-value**
Vegetation (yes%)	7 (10.1%)	6 (9.2%)	1 (25.0%)	0.355
Ejection fraction %	63 (56-65)	63.0 (56.0-65.0)	62.5 (41.3-65.0)	0.797
Aortic root (cm)	2.8 (2.8-3.1)	2.8 (2.7-3.0)	3.2 (2.9-3.3)	0.077
Left atrium (cm)	3.9 (3.5-4.3)	3.8 (3.5-4.2)	4.5 (3.7-7.5)	0.123
LV end-diastolic diam. (cm)	4.6 (4.2-5)	4.6 (4.2-5.0)	4.7 (4.5-6.2)	0.425
LV end-systolic diam. (cm)	3 (2.7-3.4)	3.0 (2.7-3.4)	3.1 (2.7-4.7)	0.579
Interventricular septum (cm)	1.2 (1.1-1.35)	1.2 (1.1-1.4)	1.2 (1.1-1.4)	0.886
LV posterior wall (cm)	1.2 (1.1-1.3)	1.2 (1.1-1.3)	1.2 (1.1-1.3)	0.906
Mitral E wave (m/s)	0.9 (0.6-1.2)	0.9 (0.6-1.2)	0.9 (0.8-1.2)	0.477
Mitral A wave (m/s)	0.9 (0.7-1.0)	0.9 (0.7-1.0)	0.7 (0.5-1.1)	0.227
Aorta velocity (m/s)	1.5 (1.3-1.8)	1.5 (1.3-1.9)	1.3 (1.2-1.6)	0.232
Aortic apex gradient (mmHg)	9.0 (6.8-12.7)	9.0 (6.8-13.0)	8.0 (7.9-10.7)	0.979
TR max velocity (m/s)	2.5 (2.3-2.9)	2.5 (2.3-2.9)	2.8 (2.3-2.9)	0.434
Pulmonary velocity (m/s)	1 (0.9-1.1)	1.0 (0.9-1.1)	1.0 (0.8-1.2)	0.902
Segmental movement defect (yes%)	14 (20.6%)	13 (20.0%)	1 (25.0%)	1.000
Pericardial effusion (yes%)	18 (26.1%)	17 (26.2%)	1 (25.0%)	1.000
Pulmonary pressure (mmHg)	37 (33-45)	37.0 (33.0-44.5)	45.0 (33-45)	0.601

Table S3. Univariable binary logistic regression analysis for 28-day all- cause mortality

**Variable**	**Univariable Analysis** OR (95% CI)	***p***	**Multivariable Analysis** OR (95% CI)	***p***
Age	1.060 (1.012–1.11)	0.014	1.055, 1.005–1.107	0.029
